# CD73 expression as a resistance mechanism in advanced *EGFR-*mutated non-small cell lung cancer

**DOI:** 10.3389/fonc.2026.1755595

**Published:** 2026-03-03

**Authors:** Inger Johanne Zwicky Eide, Anne Pernille Harlem Dyrbekk, Ina Bisha, Thomas Haberichter, Sotirios Lakis, Jessica Chan, Arthur Lewis, Philip Martin, Zachary A. Cooper, Odd Terje Brustugun

**Affiliations:** 1 Section of Oncology, Drammen Hospital, Vestre Viken Hospital Trust, Drammen, Norway; 2 Section of Cancer Genetics, Institute of Cancer Research, Oslo University Hospital, Oslo, Norway; 3 Inst of Clinical Medicine, University of Oslo, Oslo, Norway; 4 Dept of Pathology, Vestfold Hospital Trust, Tønsberg, Norway; 5 Oncology R&D, AstraZeneca, Munich, Germany; 6 Biopharmaceuticals R&D, AstraZeneca, Cambridge, United Kingdom; 7 Oncology R&D, AstraZeneca, Gaithersburg, MD, United States

**Keywords:** adenosine, CD73, EGFR, osimertinib, resistance mechanisms

## Abstract

**Introduction:**

The ectoenzyme CD73 induces an immune-evasive tumor microenvironment and has been proposed to be modulated by EGFR-TKI treatment. In this exploratory study, we analyzed CD73 expression and related immune markers during sequenced EGFR-TKI treatment, including osimertinib, to identify potential biomarkers for CD73-based therapeutic opportunities in EGFR-resistant tumors.

**Methods:**

Tumor specimens from patients included in a clinical trial (NCT02504346) evaluating osimertinib in *EGFR*-mutated EGFR-TKI pretreated NSCLC patients were analyzed. Expression of CD73, CD39, HLA-E and NKp46 were mapped in tumor tissue from diagnosis, after progression on early-generation EGFR-TKIs and after progression on osimertinib given as next-line EGFR-therapy.

**Results:**

Samples from 51 patients were evaluable. Upon progression after first line EGFR-TKI, 25 patients had T790M-postive disease, 18 cases were negative and 8 had unknown T790M-status. CD73 and HLA-E were significantly higher expressed in epithelium, while CD39 and NKp46 showed higher expression in the stroma of the tumors. There was no significant difference in expression pattern for any marker from diagnosis to progression after first line EGFR-treatment, but tumors with non-T790M-resistance to first- or second-generation TKIs had a significantly higher level of CD73 than T790M-positive tumors before commencing osimertinib. Paired tissue samples pre- and post-osimertinib were available in only four cases, of which three cases showed increased expression of HLA-E and NKp46 after osimertinib, while 2 cases had an increase in CD73 expression.

**Conclusion:**

We demonstrated differential expression patterns among the immune markers and higher levels of CD73 in cases with non-T790M-resistance to EGFR-TKIs. Although a limited number of cases were included in these analyses, the results might point to a potential role of immune markers inducing an immunosuppressive environment and thereby contribute to development of resistance to TKIs, which in turn could have future therapeutic implications.

## 1 Introduction

The prognosis of lung cancer is much improved during the last decade, but despite progress, it is still the cancer which takes most lives yearly (1). With the advent of immunotherapy, patients with advanced or metastatic non-small cell lung cancer (NSCLC) may now achieve durable responses and extended overall survival when treated with an immune checkpoint inhibitor (ICI) in monotherapy or in combination with chemotherapy in first line (2–5). However, not all patients benefit from immunotherapy. Oncogenic driven tumors with mutations in the gene encoding the epidermal growth factor receptor (EGFR) seem particularly irresponsive to checkpoint inhibitors (6, 7). Thus, first line treatment for advanced or metastatic disease is targeted treatment with EGFR tyrosine kinase inhibitors (EGFR-TKIs). Modern EGFR-TKIs are superior to older generation drugs in terms of progression free survival (PFS) and overall survival (OS) (8, 9), but resistance develops in virtually all patients. Osimertinib, a third generation TKI is currently the first choice in many countries globally. However, it was originally developed as a second line drug to target the *EGFR* T790M resistance mutation. Studies of osimertinib in the second line setting have demonstrated a PFS of around 10 months when T790M is detected at re-biopsy at progression on earlier generation EGFR-TKIs (10). Furthermore, there are indications that osimertinib to some extent exerts activity also in T790M-negative cases resistant to previous EGFR-TKIs (11).

Osimertinib is also highly active in the first line setting in the presence of sensitizing mutations (deletions in exon 19, del19, or the point mutation L858R in exon 21), yielding prolonged PFS of around 18 months when given in monotherapy and 25.5 months as combinatory treatment with chemotherapy (8, 12). However, given the poor responsiveness to immunotherapy, treatment options for *EGFR*-mutated NSCLC are limited when the disease becomes resistant to the available targeted therapies. Extensive efforts have been made to map resistance mechanisms to osimertinib with the aim of identifying new targets for treatment (13–17).

The ectoenzymes CD73 and CD39 are regulators of the adenosine pathway which is involved in inducing an immune-evasive tumor microenvironment (TME). Extracellular CD39 converts ATP to ADP and AMP and CD73 converts AMP to the immune suppressant adenosine which in turn binds to the A2A adenosine receptor (18). *EGFR*-mutated NSCLC express higher levels of CD73 compared to *EGFR* WT tumors (19) and upregulation of CD73 has been proposed as a resistance mechanism to EGFR-TKIs (20). In a phase I study, oleclumab, an antibody targeting CD73, displayed some clinical activity when given in combination with osimertinib to patients with T790M-negative disease after progression on first- or second-generation EGFR-TKIs (21), however the role of CD73-inhibition is still unclear.

In this study, we explored the expression of CD73, CD39 and related immune markers in tissue samples from different time points in the treatment course of patients with *EGFR-*mutation positive NSCLC during several lines of EGFR-directed therapies, with the aim of identifying potential biomarkers for future CD73-based treatment options.

## 2 Materials and methods

### 2.1 Patients

The TREM-trial was a phase II, single-arm, multi-institutional clinical trial conducted in Northern Europe (NCT02504346). The trial has been reported in detail previously (11). In brief, patients in this trial had advanced or metastatic *EGFR-*mutated NSCLC and were treated with osimertinib 80 mg daily after progression on at least one previous EGFR-TKI. Patients with and without the T790M resistance mutation were included. Tumor evaluation was performed by RECIST 1.1. Treatment continued until radiological progression, death or unacceptable toxicity (11).

All patients included in the clinical trial provided written informed consent. The study was approved by the Ethic Committees and relevant regulatory authorities in each participating country and was conducted in accordance with the Declaration of Helsinki and the ICH-GCP guidelines. The clinical trial was an investigator-initiated trial with Oslo University Hospital as sponsor. Funding was provided by AstraZeneca and the South-Eastern Norway Regional Health Authority. The tissue analyses in the present subset analysis were funded and performed by AstraZeneca.

### 2.2 Tissue analyses

Archival tumor tissue from the time of diagnosis was collected, and a fresh biopsy was performed at inclusion in the TREM-trial, i.e. before commencing treatment with osimertinib as next-line treatment (pre-osi). In feasible cases, tissue was also sampled at the time of progression on osimertinib (post-osi).

Expression of CD73, CD39, HLA-E and NKp46 on tumor tissue were profiled by immunohistochemistry (IHC). IHC was performed on 4 µm tissue sections using a Ventana Discovery Ultra (Roche Tissue Diagnostics) automated IHC staining platform using primary antibodies for CD73 (clone EPR6115, ab124725, Abcam, UK), CD39 (clone EPR20461, ab223843, Abcam, UK), HLA-E (clone MEM-E/02, ab2216, Abcam, UK) and NKp46 (clone MOG-1-M-H46-2/3-8E5B-5G, Innate Pharma, France), as previously described (22, 23). IHC was undertaken on the Leica Bond III IHC automated staining platform. IHC binding was demonstrated using DAB chromogenic detection followed by counterstaining with Hematoxylin. Following IHC, slides were dehydrated and cover-slipped in permanent mountant. All stained slides were reviewed for quality control by a pathologist prior to subsequent digital slide scanning at 20x magnification using a Leica Aperio AT2 Digital Pathology slide scanner (Leica, Milton Keynes, UK).

Furthermore, image analyses of tissue slides were performed to detect and quantify the different markers. After manual annotation of the tumor region on the digital images was performed by a pathologist, marker expression was quantified by AstraZeneca Computational Pathology (formerly Definiens AG) in Munich (Germany) via image analysis using Definiens Developer™ software. Customized algorithms were applied, providing readouts as percentage of positive area (of different intensities) for CD73, CD39 and HLA-E, and as densities (cells/mm^2^) for NKp46.

Expression in different regions of the tumor was explored for all markers. To separate tumor epithelium (TC-TE) from rest (tumor non-epithelium (TC-NE)) within the tumor center (TC), deep learning models were employed.

Last, the expression of all markers at different timepoints was correlated with clinical data such as progression-free survival (PFS) and overall survival (OS).

### 2.3 Statistical analyses

Expression levels of the different markers were compared with a paired Wilcoxon test. Progression free-survival and overall survival were analyzed by the Kaplan-Meier method. Groups were compared with the log rank test. For multivariate analyses, Cox regression was performed. For all analyses, a two-sided p-value under 0.05 was regarded statistically significant. As this was an exploratory study, no correction for multiple testing was performed. IBM SPSS Statistics for Windows, Version 29.0 (Armonk, NY: IBM Corp.) was used for all analyses.

## 3 Results

### 3.1 Patients

Tissue biopsies from a total of 51 of the 199 patients in the TREM-trial were included in the present biomarker study. At the time of commencement of osimertinib treatment (pre-osi), the median age of the 51 patients included in this study was 69 years and 77% were female (**Table 1**). Brain metastases were detected in 26% of the patients. The most common activating *EGFR-*mutation was deletion in exon 19 (del19) (in 53% of the patients). The T790M resistance mutation was identified in 49% of the patients, whereas 35% was T790M-negative in tissue. T790M-status was unknown in 16% of the cases due to either insufficient amount of tissue material available or no re-biopsy done before commencement of osimertinib therapy (pre-osi) (11).

**Table 1:** Baseline characteristics.

Clinical characteristics pre-osimertinib, n=51	N (%)
Median age (range) – years Mean age - years	69 (33-86) 65.8
Sex	
Male Female	12 (23.5%) 39 (76.5%)
Smoking history	
Never-smoker Former smoker Current smoker	33 (64.7%) 16 (31.4%) 2 (3.9%)
ECOG status	
ECOG 0 ECOG 1 ECOG 2	13 (25.5%) 30 (58.8%) 7 (13.7%)
Histology	
Adenocarcinoma Squamous cell carcinoma	50 (98%) 1 (2%)
Disease classification	
Stage IV	51 (100%)
*EGFR*-mutation before first line TKI treatment	
del19 Exon 20 insertion L858R L861Q	29 (56.9%) 2 (3.9%) 19 (37.3%) 1 (2.0%)
*EGFR-*mutation at start osimertinib	
del19 L858R L861Q T790M Unknown	27 (52.9%) 15 (29.4%) 1 (2.0%) 25 (49.0%) 8 (15.7%)
Brain metastases	
Yes No	13 (25.5%) 38 (74.5%)
Lines of systemic cancer treatment before osimertinib	
1 line 2 lines ≥3 lines	22 (43.1%) 12 (23.5%) 17 (33.3%)

### 3.2 Expression landscape

**Figure 1:**
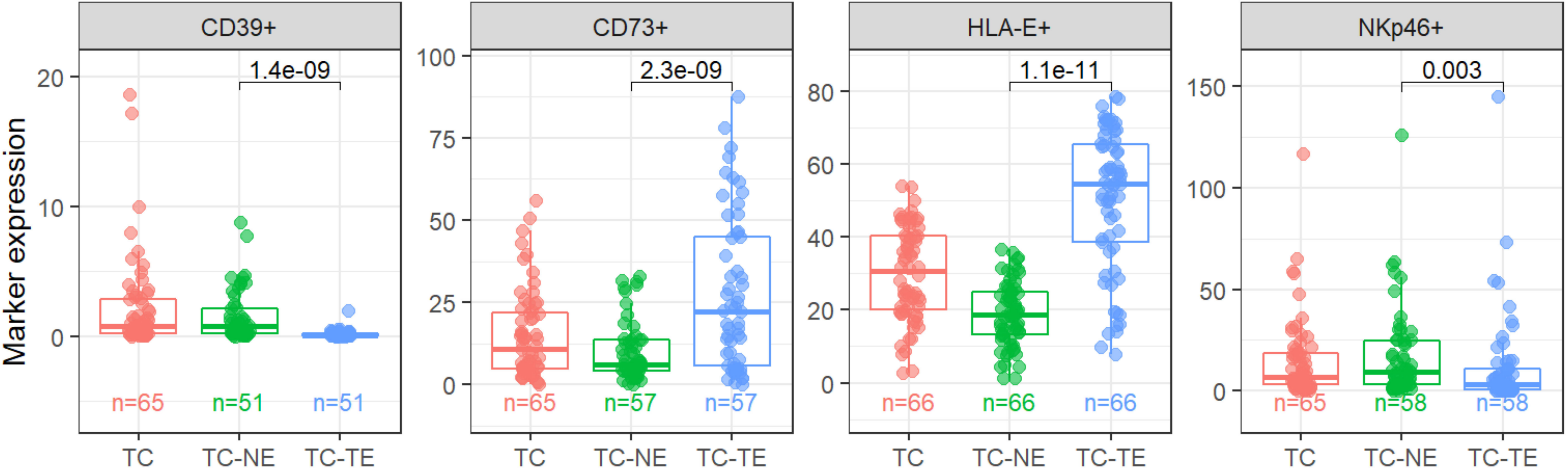
Expression levels in different regions. CD39, CD73 and HLA-E as percent of positive area, NKp46 as cell density (cells/mm2). TC, Tumor Center; TC-TE, Tumor Epithelium; TC-NE, Tumor non-Epithelium. Significant p-values indicated in the panels.

There was a significantly higher expression of CD73 and HLA-E in tumor epithelium than in stroma (non-epithelium), p<0.001 for both markers, whereas CD39 and NKp46 were expressed higher in the stroma (p<0.001 and p=0.003, respectively) (**Figure 1**). CD39 showed low expression levels in general compared to the other markers.

**Figure 2:**
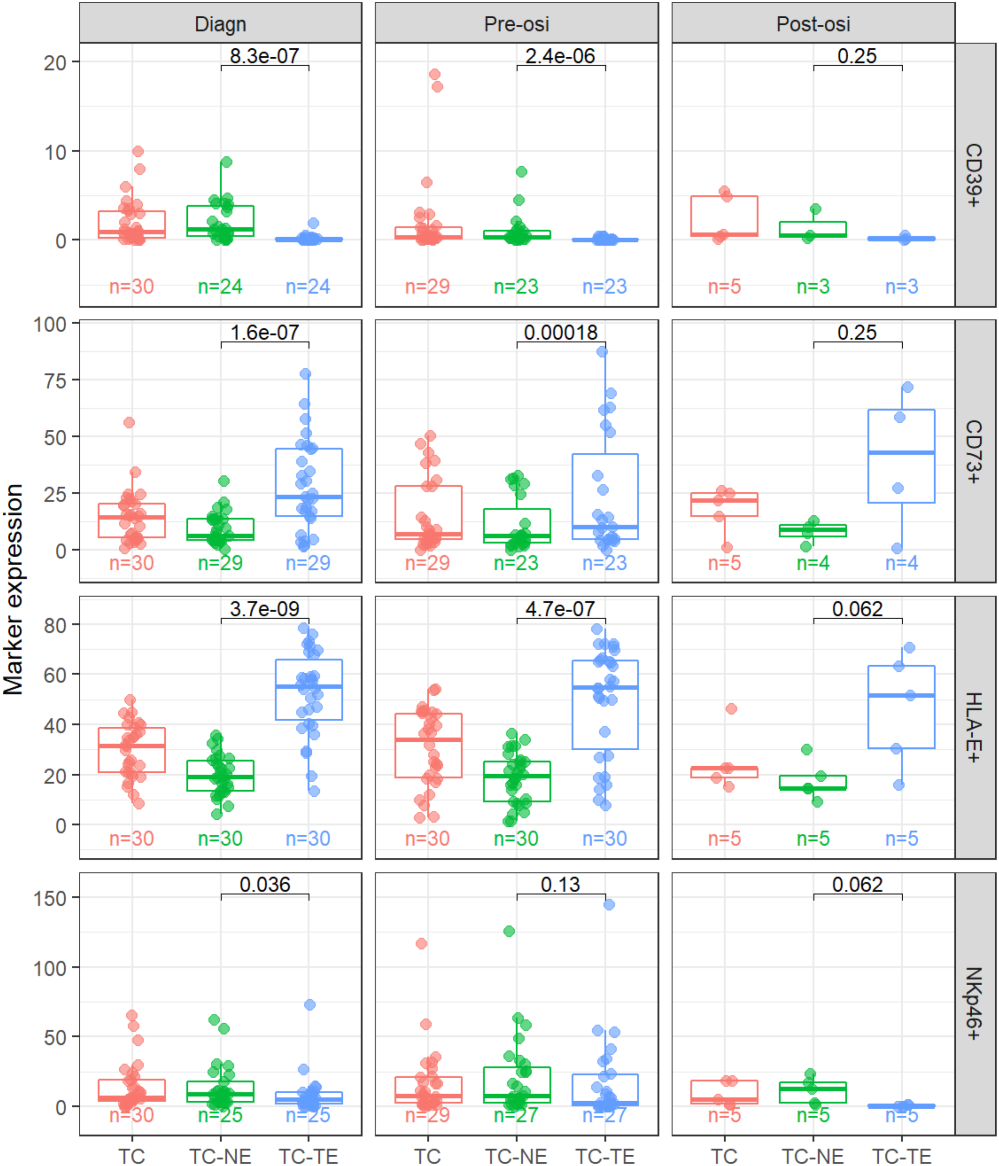
Expression levels in different regions and at different time points. CD39, CD73 and HLA-E as percent of positive area, NKp46 as cell density (cells/mm2). TC, Tumor Center; TC-TE, Tumor Epithelium; TC-NE, Tumor non-Epithelium. P-values indicated in each panel.

Of the 51 patients, 34 had samples available for analysis at the time of diagnoses, 33 at the time of start of osimertinib treatment (pre-osi) and six at the time of progression on osimertinib (post-osi). Paired pre-osi and post-osi samples were available in only four cases. The expression patterns in the different tumor regions were consistent when we analyzed expression levels at the different treatment time points (**Figure 2**).

**Figure 3:**
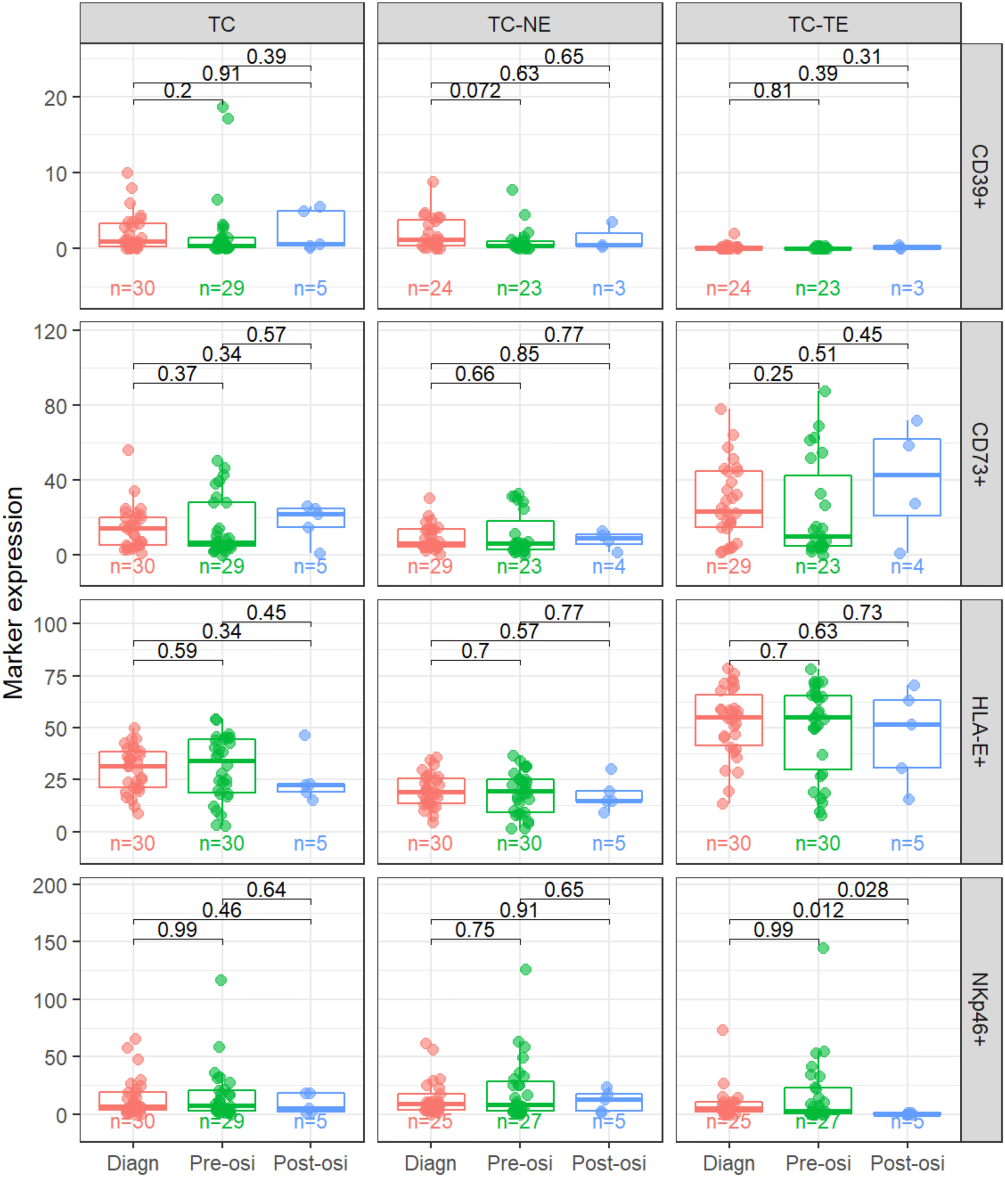
Comparison in expression levels between time points. CD39, CD73 and HLA-E as percent of positive area, NKp46 as cell density (cells/mm2). TC, Tumor Center; TC-TE, Tumor Epithelium; TC-NE, Tumor non-Epithelium; Diagn, Time of diagnosis of lung cancer; Pre-osi, Time of start of treatment with osimertinib, after progression on at least one line of EGFR-TKI; Post-osi, Time of progression on osimertinib. P-values indicated in each panel.

We next compared expression levels of the four markers between the different time points (**Figure 3**). For CD39, there was a trend for lower stromal expression pre-osi compared to a diagnosis. There were no significant differences in expression levels between diagnosis and pre-osi for CD73 nor HLA-E. However, the epithelial levels of NKp46 were significantly lower in post-osi samples compared to pre-osi and diagnostic samples.

**Figure 4:**
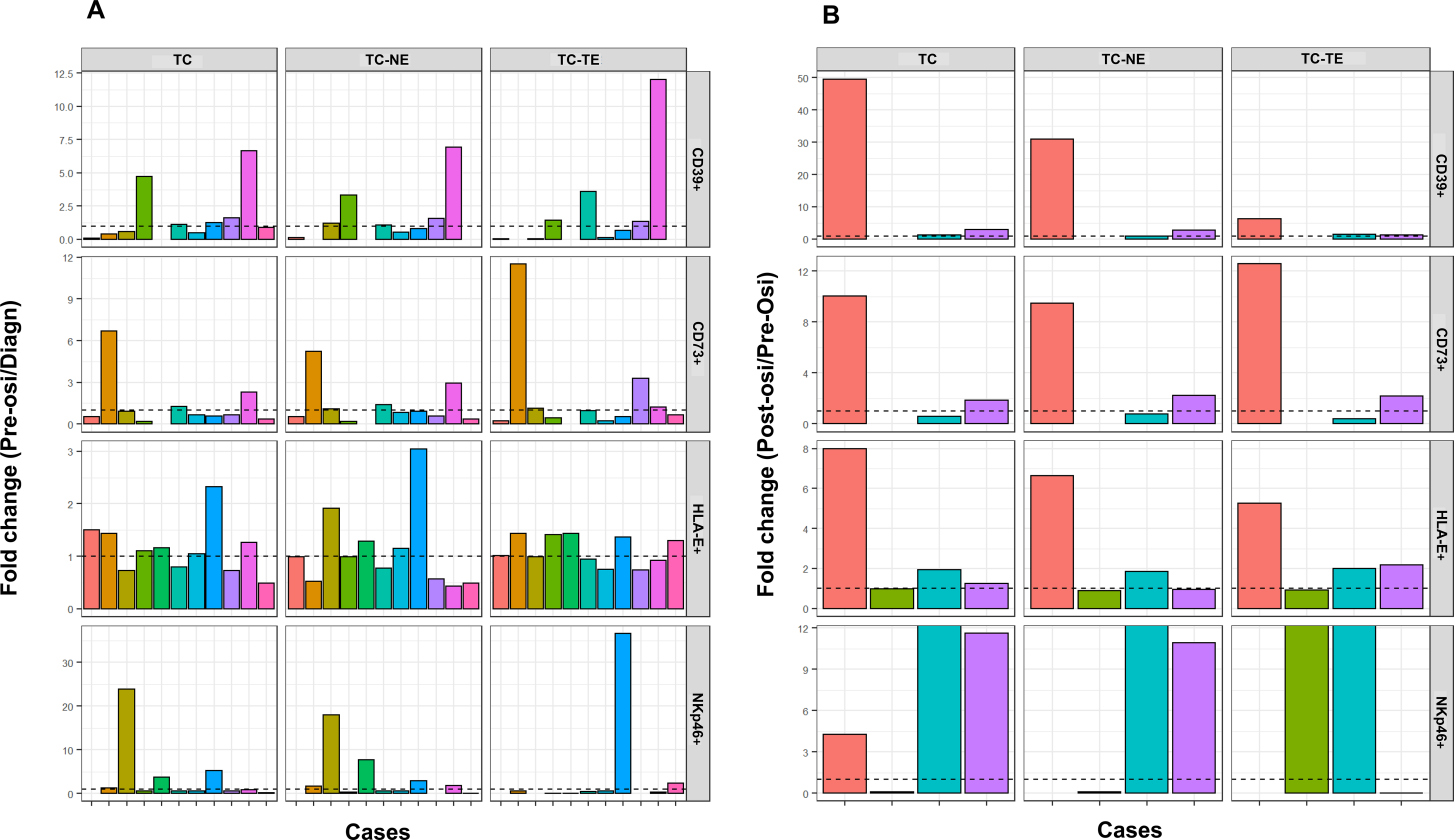
Fold changes in expression levels for paired samples. **(A)** pre-osi/diagnosis (n=11) **(B)** post-osi/pre-osi (n=4). Dashed horizontal line refers to fold change = 1, i.e. no change in marker expression for the time points under consideration.

We then investigated changes in expression levels for paired samples (**Figure 4**). No clear trends were identified for diagnosis to pre-osi expression of any of the markers. Tissue at both pre-osi and post-osi was available in only four cases. In three of these four cases there was an increase in the expression of HLA-E and NKp46, while two out of four had an increase in CD73. In one case all markers increased at the time of progression on osimertinib.

### 3.3 Correlation between expression of markers and clinical outcome

There was a significantly higher CD73-expression in samples from T790M-negative vs T790M-positive cases after first-line EGFR-therapy both in univariate (p=0.022) and multivariate (p=0.033) analysis including all markers, based on total CD73 expression at the tumor center (TC). The same trend was seen when looking at the different parts of the tumor center separately, (tumor epithelium and tumor non-epithelium, respectively) although not statistically significant. No significant differences in expression were found for the others markers with regard to T790M-status.

Overall, median time from diagnosis to start of osimertinib was 19.5 months for all patients, and in this period 57% of the patients received more than one line of systemic treatment including first- or second-generation EGFR-TKIs. Median PFS on osimertinib for all 51 patients was 7.3 months (95% CI 4.34-10.3). For patients with T790M-positive disease, median PFS was 10.8 months vs 1.6 months for patients with T790M-negative disease (HR 0.57, 95% CI 0.29-1.14, p=0.106). Median overall survival from start of osimertinib-treatment was 16.5 months (95% CI 11.7-21.3) for all patients, and 24.1 vs 11.5 months (HR 0.43, 95% CI 0.21-0.89, p=0.028) for T790M-positive vs -negative, respectively.

There was no correlation between the expression of the different markers at diagnosis and time on treatment from diagnosis to start of osimertinib. However, patients who developed T790M-positive disease after progression on previous EGFR-TKIs and had a high expression (above median) of either CD73, CD39, HLA-E or NKp46 in the pre-osi biopsy specimen, showed a trend towards shorter PFS when treated with osimertinib than the patients with biopsies with low expression (under median) of any of the markers (**Table 2**). Contrary, the patients with T790M-negative disease, had a longer PFS with high expression of either CD73 or CD39 before commencing osimertinib whereas the median PFS was shorter with high expression of HLA-E or NKp46. We also explored the 25-percentile and 75-percentile as cutoff-points for “high” vs “low” expression of the markers reported in **Table 2**, and the results were consistent (data not shown).

**Table 2:** Median PFS (months) on osimertinib for high vs low expression of the different markers. Statistically significant results are marked with asterisks.

Expression levels in biopsies at resistance to first- or second-generation EGFR-TKIs	T790M pos mPFS (95% CI)	T790M neg mPFS (95% CI)
CD73 high vs low	5.7 (0.1-11.3) vs 10.8 (4.4-17.2)	7.2 (2.2-12.2) vs 0.8 (0.3-1.3)
CD39 high vs low	5.7 (NE-11.5) vs 13.2 (8.8-17.7)	15.4 (NE-32.9) vs 1.4 (NE-6.8)*
HLA high vs low	5.7 (NE-13.7) vs 10.8 (2.6-19.0)	1.0 (0.2-1.7) vs 13.8 (3.9-23.8)
NKp46 high vs low	5.7 (NE-12.7) vs 10.8 (0.7-20.9)	1.4 (0.3-2.5) vs 16.2 (14.6-17.7)**

*p=0.042.

**p=0.006.

Patients with a low expression of CD73 in their diagnostic biopsy showed a trend towards longer overall survival than patients with high expression (59.0 months vs 36.1 months), although the difference was not statistically significant (HR 0.55, 95% CI 0.21-1.46, p=0.171). Correspondingly, similar results were seen for patients with low expression of CD39 at baseline with a median OS of 59.0 months vs 36.2 months for patients with high expression, respectively (HR 0.55, 95% CI 0.21-1.44, p=0.087). For reference, the median overall survival for all 51 patients included in this study regardless of expression of biomarkers, was 40.7 months (95% CI 31.8-49.6).

## 4 Discussion

In this study we investigated the expression of CD73 and related immune markers in tumor tissue at different time points during the disease course of patients with *EGFR*-mutated NSCLC, including across different lines of treatment with EGFR-TKIs. All patients received osimertinib after progression of first- or second line EGFR-TKIs, regardless of resistance mechanism (11). In the specimens from the patients included in this biomarker study, we found a differential expression pattern of the various markers, with high expression in tumor epithelium of CD73 and HLA-E, whereas CD39 and NKp46 were higher expressed in the stroma. This expression pattern was consistent at different time points in the sequence of treatment. As only four patients had paired samples pre- and post-osimertinib, conclusions regarding the temporal expression pattern during osimertinib treatment cannot be drawn. However we observed that two out of the four had an increase in CD73 expression and three of four had an increase in both NKp46 and HLA-E upon osimertinib exposure.

The adenosine pathway regulating ectoenzyme CD73 is expressed in many cancer types including lung cancer and is known to induce an immune-evasive TME, but also contribute to tumor progression and metastasis (24). Within the different types of lung cancers, the expression of CD73 is higher in TTF1 positive adenocarcinomas and especially *EGFR*-mutated lung adenocarcinomas (25). Interestingly, we found that the expression of CD73 was significantly higher in *EGFR-*mutation positive tumor specimens with non-T790M resistance to first- and second-generation EGFR-TKIs than in specimens with the T790M-mutation. To our knowledge, this has previously not been shown. The high expression in T790M-negative cases might suggest increased CD73 as a resistance mechanism to EGFR-directed drugs, possibly mediated through several inhibitory effects to the immune response, or more directly via interference with the EGFR signaling pathway. CD73-induced excess adenosine production leads to less effective antitumor activity by affecting a number of different immune cells. Adenosine signaling via the A2A receptor inhibits activation of T cells and stimulates the expression of other co-inhibitory receptors like PD-1 (26, 27). Likewise, dendritic cells are modulated by adenosine exposure to engage in pro-tumor activity (28, 29). Furthermore, there is evidence suggesting an interplay between adenosine levels and the activity of natural killer (NK) cells. Some studies demonstrate dysregulation of NK cells by increased levels of adenosine and even induction of CD73 on NK cells by tumor cells leading to NK cell dysfunction which affects their proliferation, cytotoxic effect and cell trafficking (30–33). Previous studies have shown that CD73 and CD39 are involved in resistance to chemotherapy by suppressing the adaptive immune response (34, 35), further supporting the hypothesis that CD73 expression might lead to resistance to other types of cancer therapies. In addition, some studies indicate a more direct effect of CD73 on EGFR signaling by cross-activation of EGFR through which resistance to EGFR-TKIs could evolve (36, 37) and also through altered phosphorylation of the EGFR by CD73 (20). Moreover, a preclinical study demonstrated that MET-amplification upregulate CD73 in EGFR-TKI resistant cells, creating a tumor-promoting TME and thus providing another possible explanation for the link between CD73 expression and therapy resistance (38).

As CD73 expression has been shown to be associated with poor prognosis in NSCLC (25, 39), we speculate that the high expression in T790M-negative cases in our study might contribute to the modest PFS to osimertinib for these patients in second or later lines (11). Additionally, we also found a trend towards shorter overall survival from the time of diagnosis and irrespective of number of treatment lines for patients with high expression of CD73 in their diagnostic biopsy, supporting the notion that CD73 is a prognostic factor.

Intriguingly, in our study, patients with non-T790M-resistance to EGFR-TKIs and high expression of NKp46 before commencing osimertinib as next-line treatment had a shorter PFS than patients with low expression of NKp46. The reason for the observation of less clinical benefit of osimertinib despite the abundant presence of NK cells is not known, however, a possible explanation is the adenosine-induced dysregulation of NK cells, as discussed above (33). However, patients with high expression of CD73/CD39 at start of osimertinib treatment showed at trend towards a larger clinical benefit of the drug in terms of PFS.

Preclinical studies have identified the CD73/adenosine pathway as a potential therapeutic target to sensitize *EGFR-*positive tumors to immunotherapy (40). In a phase I study investigating the combination of the CD73 antibody oleclumab and durvalumab, 9.5% of 42 patients with *EGFR* -mutated NSCLC achieved a partial response and 21% had stable disease (41). There have also been different attempts of targeting the CD73/adenosine pathway without including immune checkpoint inhibitors. Oleclumab demonstrated promising activity in combination with osimertinib in patients with T790M-negative disease after first- or second-generation TKIs (21). Among other potentially relevant agents are the MEK1/2 inhibitors selumetinib and PD318088, as for these a correlation of drug sensitivity and the protein levels of CD73 has been shown (39). Nevertheless, there is still a large unmet need to develop effective treatment options for patients with *EGFR-*mutated NSCLC resistant to established treatments and thus also a need for identifying biomarkers to guide the development of new drugs and patient selection.

The treatment landscape of *EGFR*-mutated NSCLC has been rapidly moving forward, and this study was designed when first- or second-generation EGFR-TKIs were standard of care as first line treatment. However, increasing knowledge of clinical activity and resistance mechanisms to osimertinib given in later lines might still be of relevance as many patients, especially in some territories, still receive sequenced EGFR-directed therapies. Furthermore, as upregulation of CD73 is known to contribute to modulation of the TME and immune evasion in multiple cancers and in different treatment settings, there is a rationale to investigate the role of CD73 within the present treatment algorithm for *EGFR*-mutated NSCLC at resistance to osimertinib given as first line treatment. The FLAURA-2-study demonstrated superior efficacy of osimertinib in combination with chemotherapy in the first line setting (12), and some retrospective studies also suggest that adding chemotherapy to osimertinib at the time of progression on osimertinib monotherapy might be of clinical benefit for patients (42). Furthermore, the MARIPOSA-trial recently demonstrated an overall survival benefit in favor of combination therapy with the bispecific MET/EGFR-antibody amivantamab and a third-generation EGFR-TKI compared to osimertinib as monotherapy (43). The different combination therapies come with an increased burden of toxicity, and how to best select which patients who benefit from treatment enhancement is still unclear. Future studies should be done to elucidate whether CD73 could be utilized as a marker for not only resistance to treatment, but also to guide upfront treatment. Therefore, it is of great importance that clinical studies are designed to incorporate well conducted translational analyses to identify biomarkers for patient selection in the choice of treatment at different stages of the disease.

There are several limitations to this work. Progression samples were scarce, and thus the sample size small, precluding firm interpretation of the results. We did not have any information on PD-L1-expression which could be relevant in the context of evaluating the immunogenicity of the tumors, especially as data regarding the role of PD-L1 in *EGFR*-mutant NSCLC are conflicting (44, 45). Furthermore, more extensive analyses to reveal other resistance mechanisms to TKI-treatment at the different time points could have been informative. Taken together, this study is descriptive and should be considered hypotheses generating, stimulating further research in this field.

In conclusion, in this explorative biomarker study we found that CD73 is expressed in higher degree in tumor samples with non-T790M-mediated resistance after treatment with first- or second-generation EGFR-TKIs. Although the study design and limited sample size do not allow for causality assessments, these findings contribute to the concept of possible involvement of immune markers in the development of resistance to EGFR-TKIs. Further research to pursue the identification of resistance mechanisms, predictive biomarkers and efforts to enhance immunogenicity of *EGFR*-mutated NSCLC should be undertaken.

## Data Availability

The datasets presented in this article are not readily available because data sharing might violate data protection regulations due to clinical information on a limited number of patients. Requests to access the datasets should be directed to ingei@vestreviken.no.
